# Supplementary material for: Following replicative DNA synthesis by time-resolved X-ray crystallography
Source: Nat Commun. 2021 May 11;12:2641. doi: 10.1038/s41467-021-22937-z (PMC8113479; doi:10.1038/s41467-021-22937-z)
Supplement: Supplementary file 1 — Supplementary Information [file 41467_2021_22937_MOESM1_ESM.pdf]

Supporting Information

Following Replicative DNA Synthesis by Time-Resolved X-ray Crystallography

Nicholas Chim[1], Roman A. Meza[1], Anh M. Trinh[1], Kefan Yang[4], and John C. Chaput[1-3]*

[1]Department of Pharmaceutical Sciences, [2]Department of Chemistry, [3]Department of Molecular Biology and Biochemistry, and [4]Department of Chemical and Biomedical Engineering.
University of California, Irvine, CA 92697-3958.

This PDF includes:

Supplementary Tables 1 – 5
Supplementary Figures 1 – 5

Supplementary Table 1. Oligonucleotides used in this study.

Oligonucleotide name	Oligonucleotide sequence (5' – 3')	Comments
**Template, T**	GACGTACGTGATCGCA	Template used for crystallography
**Primer, P**	GCGATCACGT	Primer used for crystallography
**Bst_Y714S_FWD**	TGGGATCGTT*TCC*GGGATCAGTG	Q5 SDM forward primer, s*er mutation*
**Bst_Y714S_RVS**	AAGTTGACCGCCTTCGCC	Q5 SDM reverse primer
**Bst_Y719S_FWD**	GATCAGTGAT*TCC*GGCTTGGCGC	Q5 SDM forward primer, s*er mutation*
**Bst_Y719S_RVS**	CCGTAAACGATCCCAAAGTTG	Q5 SDM reverse primer
**Activity Primer**	/IR680/CGCGAACTGCG	5' IR680 primer for polymerase activity assay
**Poly-T Template**	CGTTTTTAACGCAGTTCGCG	Template for polymerase activity assay
**Poly-A Template**	GCAAAAATTCGCAGTTCGCG	Template for polymerase activity assay
**Poly-C Template**	GTCCCCCAACGCAGTTCGCG	Template for polymerase activity assay
**Poly-G Template**	CTGGGGGAACGCAGTTCGCG	Template for polymerase activity assay

Supplementary Table 2. Datasets collected for initiation cycle of DNA synthesis

Time-lapsed datasets from a single substrate (dATP) soak

Rxn time (min)	Datasets analyzed#	Predominant conformation
0.5	4	Start structure (Time = 0)
1*	7	Duplex migration
1.5	4	
1.7	2	
2	4	
3.3	4	
4*	3	Next templating base occupies hydrophobic pocket
5	3	
6	2	
8*	1	Nucleotide insertion and translocation complete
10	6	
15	2	
20	2	
30	3	
40	5	
60	8	
75	1	
90	5	
120*	6	Nucleotide addition complete
150	7	

#Total data sets analyzed 79

*Representative deposited structure

Supplementary Table 3 Initiation cycle data collection and refinement statistics

	1 min	4 min	8 min	120 min
**Data Collection**				
Space group	$P2_12_12_1$	$P2_12_12_1$	$P2_12_12_1$	$P2_12_12_1$
Cell Dimensions				
a, *b*, *c* (Å)	87.0, 93.1, 104.8	87.5, 93.3, 105.5	88.2, 94.0, 105.7	87.8, 93.2, 104.9
α, β, γ (º)	90.0, 90.0, 90.0	90.0, 90.0, 90.0	90.0, 90.0, 90.0	90.0, 90.0, 90.0
Resolution (Å)	44.89-2.16 (2.24-2.16)	38.35-1.97 (2.04-1.97)	39.92-2.0 (2.07-2.0)	46.59-2.3 (2.38-2.3)
R_{merge}	0.164 (1.047)	0.048 (0.186)	0.065 (0.30)	0.142 (0.993)
CC1/2	0.997 (0.776)	0.998 (0.959)	0.996 (0.951)	0.997 (0.786)
$I / \sigma I$	12.5 (2.6)	22.0 (10.0)	14.9 (5.5)	13.7 (2.4)
Completeness (%)	99.9 (99.8)	97.7 (99.8)	95.7 (99.9)	99.7 (99.7)
Redundancy	7.0 (5.9)	4.6 (4.9)	4.7 (4.9)	6.6 (5.5)
**Refinement**				
Resolution (Å)	2.16	1.97	2.0	2.3
No. reflections	46273 (4539)	60953 (6101)	59144 (5882)	38829 (3811)
R_{work}/R_{free}	0.186/0.227 (0.295/0.354)	0.219/0.258 (0.23/0.287)	0.194/0.246 (0.208/0.272)	0.188/0.238 (0.268/0.313)
No. atoms	5331	5793	5576	5317
Protein/DNA	5100	5125	5101	5135
dATP	-	-	21	-
Water/Sulfate	231/25	663/5	439/15	167/15
B-factors	50.9	33.2	44.9	52.5
Protein/DNA	50.9	32.9	44.4	52.6
dATP	-	-	104.3	-
Water/Sulfate	46.3/95.3	32.9/58.3	46.6/84.4	46.2/110.1
R.m.s deviations				
Bond lengths (Å)	0.008	0.008	0.008	0.008
Bond angles (º)	1.19	1.19	1.17	1.2

*Values in parentheses are for the highest-resolution shell.

Supplementary Table 4. Datasets collected for the elongation cycle of DNA synthesis

Time-lapsed datasets from simultaneous double substrate (dATP and dCTP) soak

Rxn time (hr)	Datasets analyzed#	Predominant conformation
2	5	1st nucleotide addition (dATP) complete†
3	4	Next templating base occupies hydrophobic pocket
3.5	1	
4*	13	
5	7	
6	6	
8	1	
14	1	
24	10	
25.5*‡	1	Nucleotide (dCTP) insertion and translocation complete
27‡	2	
48*	3	2nd nucleotide addition (dCTP) complete

#Total data sets analyzed 54

*Representative deposited structures

†Similar conformation to structures from initiation cycle, reaction time 120 min

‡Datasets derived from sequential substrate soak (1st: dATP, 2nd: dCTP)

Supplementary Table 5. Data collection and refinement statistics for the elongation cycle of DNA synthesis

	4 hr	25.5 hr	48 hr
**Data Collection**			
Space group	$P2_12_12_1$	$P2_12_12_1$	$P2_12_12_1$
Cell Dimensions			
a, *b*, *c* (Å)	86.6, 93.4, 105.9	86.6, 93.6, 105.5	87.3, 93.5, 105.2
α, β, γ (º)	90.0, 90.0, 90.0	90.0, 90.0, 90.0	90.0, 90.0, 90.0
Resolution (Å)	46.05-1.67 (1.73-1.67)	46.79-2.3 (2.38-2.3)	45.06-2.0 (2.07-2.0)
R_{merge}	0.055 (0.807)	0.062 (0.480)	0.064 (0.164)
CC1/2	0.999 (0.786)	0.999 (0.932)	0.997 (0.959)
$I / \sigma I$	18.4 (2.3)	19.2 (3.8)	20.3 (10.5)
Completeness (%)	98.1 (98.4)	99.8 (99.9)	98.2 (97.7)
Redundancy	6.5 (6.8)	6.5 (6.8)	6.3 (6.2)
**Refinement**			
Resolution (Å)	1.67	2.3	2.0
No. reflections	99198 (9743)	38767 (3819)	58065 (5681)
R_{work}/R_{free}	0.187/0.210 (0.246/0.285)	0.191/0.237 (0.238/0.331)	0.190/0.23 (0.246/0.289)
No. atoms	5720	5257	5639
Protein/DNA	5142	5106	5125
dCTP	-	16	-
Water/Sulfate	581/15	120/15	499/15
B-factors	38.1	57.5	35.3
Protein/DNA	37.4	57.5	34.8
dCTP	-	90.4	-
Water/Sulfate	43.2/83.1	47.5/107.6	38.6/83.6
R.m.s deviations			
Bond lengths (Å)	0.007	0.008	0.008
Bond angles (º)	1.11	1.16	1.13

*Values in parentheses are for the highest-resolution shell.

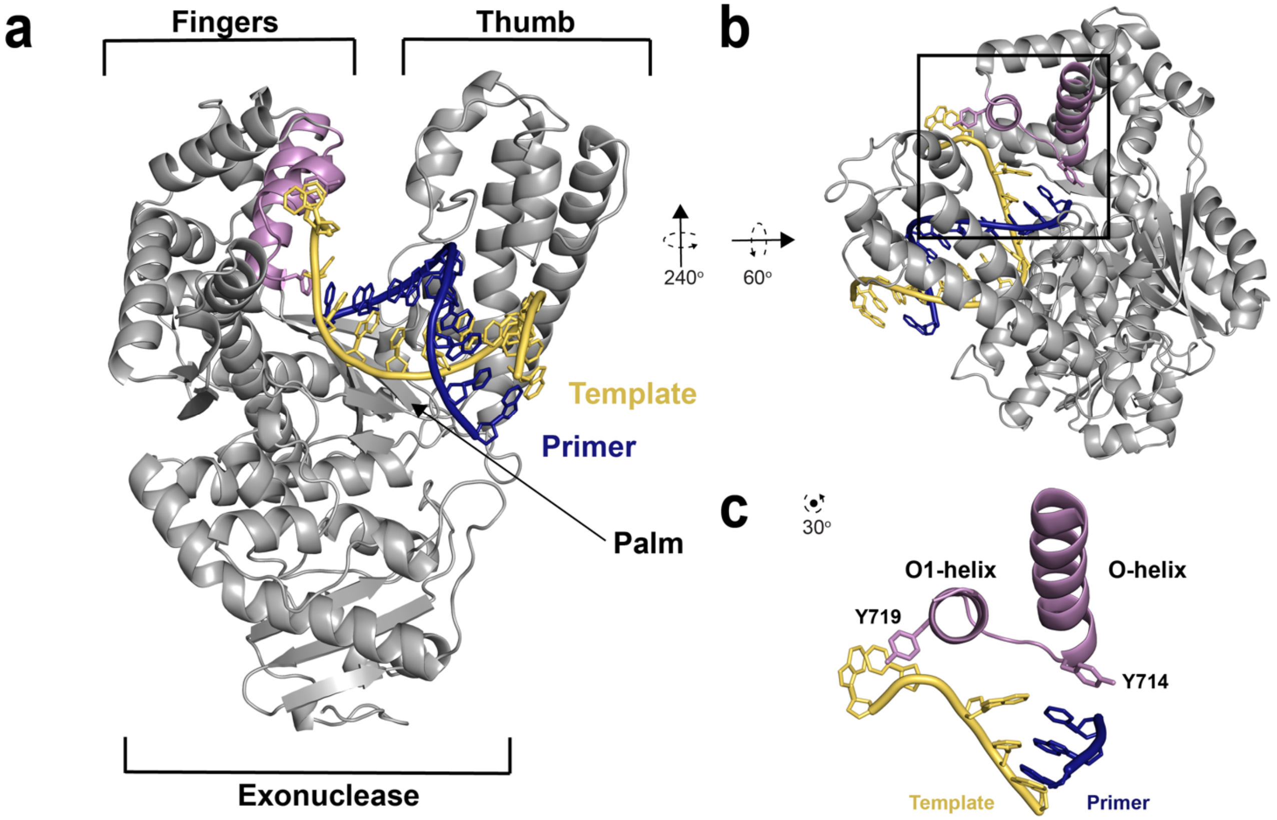

Supplementary Figure 1. Crystal structure of Bst DNA polymerase. (**a**) Global architecture of Bst bound to the primer-template duplex. (**b**) Rotated view revealing Bst active site (boxed). (**c**) Zoomed-in view of the active site highlighting the primer-template duplex, the O and O1 helices and their respective conserved tyrosine residues, Y714 and Y719. The zoom-in view is represented throughout the manuscript, since it is this region of the protein that undergoes conformational changes during the catalytic cycle. Color scheme: primer (blue), template (yellow), and O and O1 helices of the finger subdomain (purple).

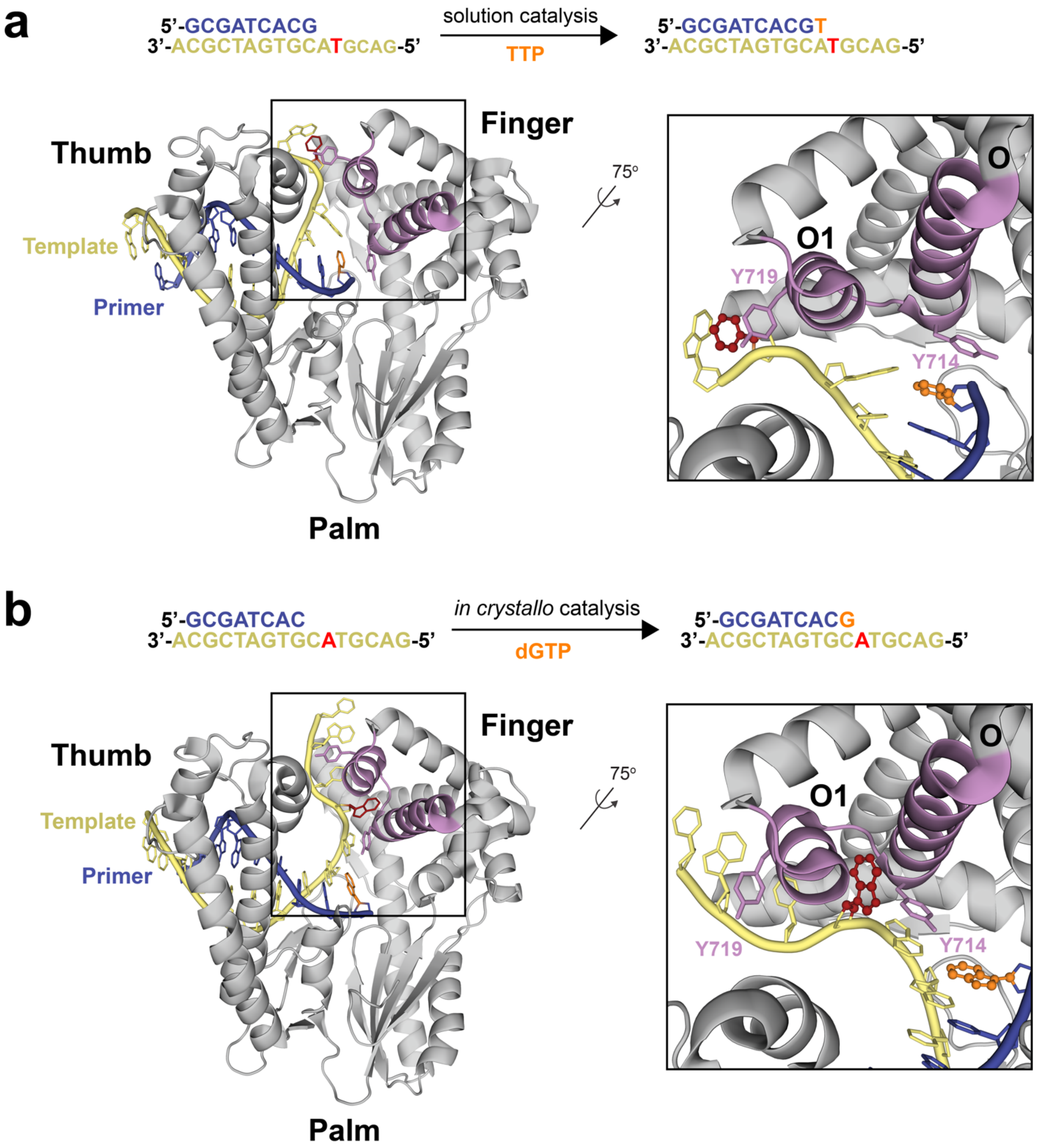

Supplementary Figure 2. Crystal structures of Bst DNA polymerase capturing different intermediates in the reaction pathway. The polymerase and active site region for a primer extension reaction performed (**a**) in solution and crystallized (PDB: 6DSY) and (**b**) performed in crystallo via soaking (PDB: 1L3S). Note the differences in positioning of the next templating base (red, ball and stick) and Y714 residue. The vestigial exonuclease domain (residues 297 – 492) is omitted for clarity. Color scheme: primer (blue), template (yellow), incoming dNTP (orange, ball and stick), and O and O1 helices of the finger subdomain (purple).

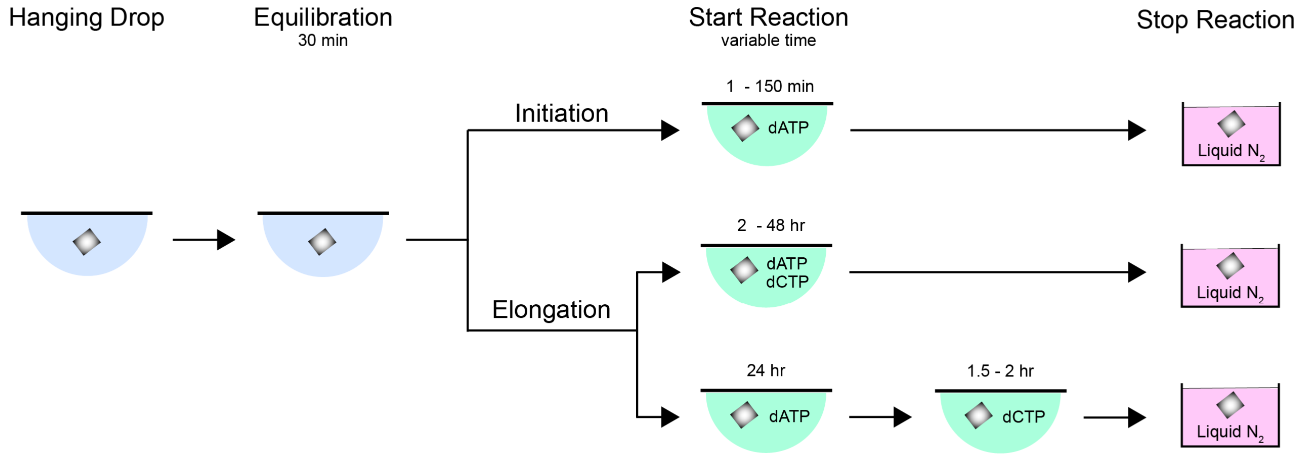

Supplementary Figure 3. Strategy for time-resolved X-ray crystallography. Crystals of the binary enzyme-DNA complex grown in standard hanging drop format were harvested and transferred to an equilibration buffer for 30 minutes to remove excess crystallization reagents. The polymerase reactions were initiated by transferring the crystal from the equilibration solution to a reaction solution containing the necessary dNTP substrate(s). At designated times, the reactions were stopped by plunging the crystals into liquid nitrogen. Frozen crystals were evaluated by X-ray crystallography. For the initiation cycle, crystals were transferred to reaction droplets containing dATP, while the elongation cycle required transferring crystals to reaction droplets containing both dATP and dCTP. The structure obtained for the 25.5-hour time point in the elongation cycle was obtained using a sequential strategy that involved incubation with dATP followed by a second incubation with dCTP.

a

```
IR680-CGCGAACTGCG
      GCGCTTGACGCAATTTTTGC        T-Template

IR680-CGCGAACTGCG
      GCGCTTGACGCTTAAAAAGC        A-Template

IR680-CGCGAACTGCG
      GCGCTTGACGCAACCCCCTG        C-Template

IR680-CGCGAACTGCG
      GCGCTTGACGCAAGGGGGTC        G-Template
```

b

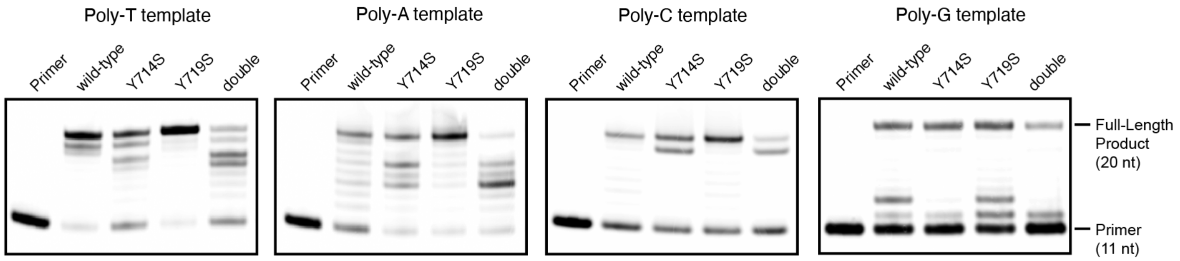

Supplementary Figure 4. Polymerase activity assay. (**a**) Nucleotide sequences of the DNA primer-template duplexes. (**b**) Analysis of the primer extension reaction by 20% denaturing polyacrylamide gel electrophoresis. Primer-extension of dNTP substrates on DNA templates by wild-type and mutant Bst DNA polymerases. Reactions were performed in ThermoPol buffer [20 mM Tris, 10 mM $(NH_4)_2]SO_4$, 10 mM KCl, 2 mM $MgSO_4$, 0.1% Triton X-100, pH 8.8] with 0.5 μM DNA primer-template duplex, 100 μM dNTPs, and 0.01 μM polymerase for 3 minutes at 50 °C. Primer-extension assays were performed in triplicate as independent experiments. Bst DNA polymerase mutants: Y714S, Y719S, and double (Y714S and Y719S).

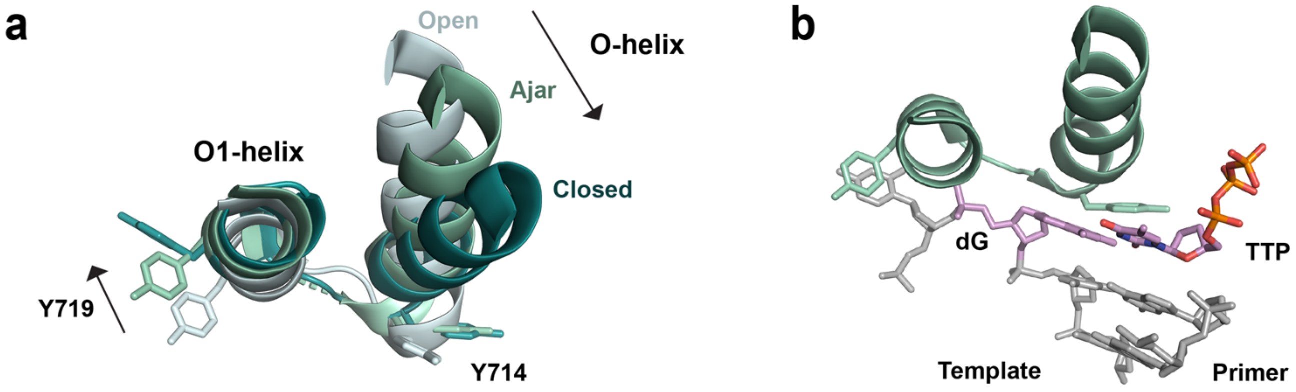

Supplementary Figure 5. Structure of a dG:TTP mismatch trapped in the ajar conformation of Bst DNA polymerase. (**a**) Structural comparison of the open (PDB: 1L3S), ajar (PDB: 3HP6), and closed (PDB: 1LV5) conformations of Bst DNA polymerase. (**b**) Structural view of the active site of the ajar conformation of Bst DNA polymerase containing a dG:ddTTP mismatch (purple).
