# Supplementary material for: Following replicative DNA synthesis by time-resolved X-ray crystallography
Source: Nat Commun. 2021 May 11;12:2641. doi: 10.1038/s41467-021-22937-z (PMC8113479; doi:10.1038/s41467-021-22937-z)
Supplement: Supplementary file 3 — Description of Additional Supplementary Files [file 41467_2021_22937_MOESM3_ESM.pdf]

Description of Additional Supplementary Files

File Name: Supplementary Movie 1
Description: Animated movie showing the initiation and elongation cycles of DNA synthesis.
